# Quantitative ultrasound imaging reveals distinct fracture-associated differences in tibial intracortical pore morphology and viscoelastic properties in aged individuals with and without diabetes mellitus – an exploratory study

**DOI:** 10.3389/fendo.2024.1474546

**Published:** 2024-12-16

**Authors:** Carolin Dehnen, Angela Galindo, Paula Hoff, Oliver Palme, Lukas Maurer, Kay Raum, Edgar Wiebe

**Affiliations:** ^1^ Department of Rheumatology and Clinical Immunology, Charité – Universitätsmedizin Berlin, Corporate Member of Freie Universität Berlin and Humboldt- Universität zu Berlin, Berlin, Germany; ^2^ Charité – Universitätsmedizin Berlin, Corporate Member of Freie Universität Berlin and Humboldt- Universität zu Berlin, Berlin, Germany; ^3^ Endokrinologikum Berlin, Medizinisches Versorgungszentrum (MVZ) am Gendarmenmarkt, Berlin, Germany; ^4^ Department of Endocrinology and Metabolic Diseases, Charité – Universitätsmedizin Berlin, Corporate Member of Freie Universität Berlin and Humboldt- Universität zu Berlin, Berlin, Germany

**Keywords:** bone, diabetes mellitus, fracture, turnover (TO), ultrasound

## Abstract

**Introduction:**

Diabetes mellitus (DM) is a chronic metabolic disorder that increases fragility fracture risk. Conventional DXA-based areal bone mineral density (aBMD) assessments often underestimate this risk. Cortical Backscatter (CortBS) ultrasound, a radiation-free technique, non-invasively analyzes cortical bone’s viscoelastic and microstructural properties. This study aimed to evaluate CortBS’s discriminative performance in DM patients compared to DXA and characterize changes in cortical bone microstructure in Type 1 and Type 2 DM (T1DM, T2DM) patients.

**Methods:**

This *in-vivo* study included 89 DM patients (T1DM = 39, T2DM = 48) and 76 age- and sex-matched controls. DXA measured aBMD, while CortBS measurements were taken at the anteromedial tibia using a medical ultrasound scanner with custom software. Multivariate analysis of variance assessed the impact of DM type on CortBS and DXA measurement results. Partial least squares discriminant analyses with cross-validation were used to compare the discrimination performance for vertebral, non-vertebral, and any fragility fractures, adjusting for gender, age, and anthropometric parameters (weight, height, BMI).

**Results:**

Fractures occurred in 8/23 T1DM, 17/18 T2DM, and 16/55 controls. DXA parameters were reduced in fracture patients, with significant diabetes impact. T2DM was associated with altered CortBS parameters, reduced scatterer density, and larger pores. CortBS outperformed DXA in discriminating fracture risk (0.61 ≤ AUC(DXA) ≤ 0.63, 0.68 ≤ AUC(CortBS) ≤ 0.69).

**Conclusions:**

Both T1DM and T2DM showed altered bone metabolism, with T2DM linked to impaired tissue formation. CortBS provides insights into pathophysiological changes in diabetic bone and provided superior fracture risk assessment in DM patients compared to DXA.

## Introduction

Diabetes mellitus (DM) is a chronic metabolic condition with high prevalence, affecting 422 million individuals worldwide (1). It is categorized into two main types: Type 1 (T1DM) and Type 2 (T2DM). While T1DM is characterized by a complete lack of insulin due to autoimmune destruction of pancreatic beta cells (2), T2DM is a multifactorial disorder with later onset, defined by insulin resistance and progressive beta cell failure, leading to a gradual decrease in insulin secretion (3). DM of both types has a detrimental effect on bone health and is associated with an increased fracture risk. T1DM shows a significantly elevated risk for non-vertebral and hip fractures (relative risks of 3.8 and 6.9, respectively) (4, 5), while T2DM shows a moderately increased risk for any fracture type (5–7). Factors, such as prolonged disease duration, insulin use (7), certain oral antidiabetic drugs, such as thiazolidines (8), and poor glycemic control (9), are associated with higher fracture incidence.

Several mechanisms contribute to bone fragility in diabetic patients, including decreased parathyroid hormone levels, hyperglycemia, accumulation of advanced glycation end products (AGEs) in tissues, and impaired osteoclast function (10–12). In T1DM, osteocyte apoptosis and micropetrosis—characterized by the mineralization of abandoned osteocyte lacunae—have been linked to the increase in microdamage and are proposed as potential contributors to impaired bone remodeling (13). A reduction in osteocyte density indicates altered cellular activity and bone quality (14), resulting in compromised cell communication with osteoblasts and osteoclasts. Their attenuated activity leads, subsequently, to the clinically observed low bone turnover (10). A meta-analysis reported decreased levels of both the bone resorption marker C-terminal cross-link of collagen (CTX) and the bone formation marker osteocalcin in T1DM compared to control individuals (15). Similarly, a lower bone turnover has been observed in postmenopausal women with T2DM compared to controls (16).

The impaired cellular function and bone turnover have an impact on the bone tissue composition and structure. At the microscopic level, inconsistent evidence exists regarding altered levels of tissue mineralization rates. Some studies show elevated and less heterogenous bone mineralization in T2DM (17), while others do not find significant abnormalities (18). In T1DM, differences in mineral maturity and crystallinity have been observed in long-standing disease (11). However, in both DM types, tissue strength deteriorates due to factors, including collage glycation and decreased cross-link strength (15).

The Haversian bone formation rate is an indicator of bone formation rates within the osteons or the cortical Haversian system. An early post-mortem histomorphometry study by Wu et al. (19) reports that individuals with DM have a decreased Haversian bone formation rate, which corresponds to only 39% of the normal rate. Haversian bone formation normally increases two-fold between ages 35 and 60 years. However, the authors calculated a reduction down to 22% with the onset of the disease. The compromised intracortical tissue remodeling results in a reduced number of pores and an increased average tissue age. Additionally, a 31% reduction in vascular canal density has recently been observed in a T2DM rat model (14).

Areal Bone Mineral density (*aBMD*) measured by Dual energy-X-ray-Absorptiometry (DXA) often underperforms in diabetic individuals (5). In T1DM, low *aBMD* in children and adolescents has been linked to insulin deficiency and lower insulin growth factor 1 (IGF-1) levels, although these differences compared to controls do not entirely account for the increased fracture risk (20). Marginal differences have been observed in middle-aged diabetic women when compared to healthy women (21). In T2DM, *aBMD* values tend to be higher compared to those without the disease (5). For any given *aBMD* level, T2DM patients have a higher fracture risk compared to non-diabetic individuals (22). This phenomenon, which overestimates bone strength and underestimates fracture risk, is known as the “diabetic paradox.” The fracture risk assessment tool FRAX underperforms similarly to DXA in both DM types (22, 23). The Trabecular Bone Score (TBS) has been introduced as an indirect measure of trabecular bone microstructure, becoming a significant addition to DXA. In both Type 1 and Type 2 DM, TBS values are lower (24, 25), implying that despite similar bone density, the trabecular bone microstructure deteriorates, contributing to increased bone fragility.

Burghardt et al. (26) have first elucidated the changed microstructure in diabetic bone using high-resolution peripheral computer tomography (HR-pQCT). The study revealed increased cortical porosity and trabecular BMD in women with T2DM compared to controls. In a following study, Patsch et al. found an association between cortical porosity and prevalent fractures in women with T2DM (27). A meta-analysis on first-generation HR-pQCT (28) confirmed that T2DM patients displayed elevated cortical porosity together with an improved trabecular microarchitecture (29). On the other hand, reduced quality of the trabecular department was observed in T1DM. A post-mortem study of the impact of microvascular disease (MVD) on bone revealed weakened trabecular and cortical compartments in T1DM patients with MVD (30). More research is needed to characterize changes in the diabetic bone structure. However, these results indicate that bone fragility in diabetic individuals is not solely determined by BMD and that devices for measuring bone microstructure could improve fracture prevention. The tibia has recently been suggested as a preferable site for measuring cortical bone, aiding in the understanding of site-specific structural and compositional factors that determine bone quality in DM (30). A wide application of HR-pQCT in clinical routine is unlikely due to its high costs and limited availability. Quantitative Ultrasound (QUS) of the bone presents a low-cost, portable and radiation-free alternative.

QUS devices have been developed for bone measurement at various anatomical sites, including trabecular bone at the calcaneus, spine, and femoral neck, and cortical bone at the radius, tibia, femur, and phalanx (31). First-generation non-imaging QUS devices measure ultrasound propagation properties, e.g. speed of sound (SOS) and broadband ultrasound attenuation (BUA), from which surrogate parameters, like stiffness index, apparent thickness, BMD, osteoporosis diagnosis, and fragility scores, were derived by means of empirical associations. New cortical bone technologies make use of sophisticated 2D or 3D refraction-corrected imaging and model-based spectral analysis of sound waves scattered at intracortical pores (31). The application of QUS in patients with DM has yielded mixed results. Studies using QUS devices of the calcaneus have shown lower QUS parameters in diabetic patients (32, 33), while others have found no differences between diabetes and controls (34) or even QUS parameters comparable to DXA T-Scores (35, 36). Recently, the application of the radiofrequency-echographic-multispectrometry (REMS), which analyses ultrasound backscatter of trabecular tissue at the femoral neck and spine, revealed lower REMS-BMD and elevated DXA T-Scores in T2DM women (37).

Cortical Backscatter (CortBS) is an innovative bone QUS imaging technology that assesses viscoelastic and microstructural properties at the anteromedial tibia shaft (38). By employing 3D ultrasound measurement and spectral analysis of sound waves backscattered from intracortical pores, the intracortical pore size distribution is determined. Notably, this spectral approach allows for the quantification of pore sizes as small as 20 µm, exceeding the physical resolution limit of HR-pQCT, which is approximately 90 µm (38). A pilot study involving postmenopausal women with low BMD demonstrates that the discrimination performance of this technique is comparable to that of HR-pQCT and superior to DXA (39). Given the increasing evidence that bone fragility is caused by reduced cortical bone quality, we hypothesized that CortBS could serve as a promising alternative diagnostic tool. It is expected that CortBS would be particularly sensitive to the increased porosity in T2DM patients and may further aid in depicting the structural changes in the cortical bone of individuals with DM.

This cross-sectional study, therefore, aimed to apply the CortBS method for the first time in men and women with T1DM and T2DM compared to controls without DM. The primary objective was to compare CortBS to the gold-standard DXA and evaluate the discrimination performance of both methods for prevalent fractures. The secondary objectives were to evaluate differences in cortical bone microstructure with respect to fractures and DM type.

## Materials and methods

### Study design and participants

**Figure 1:**
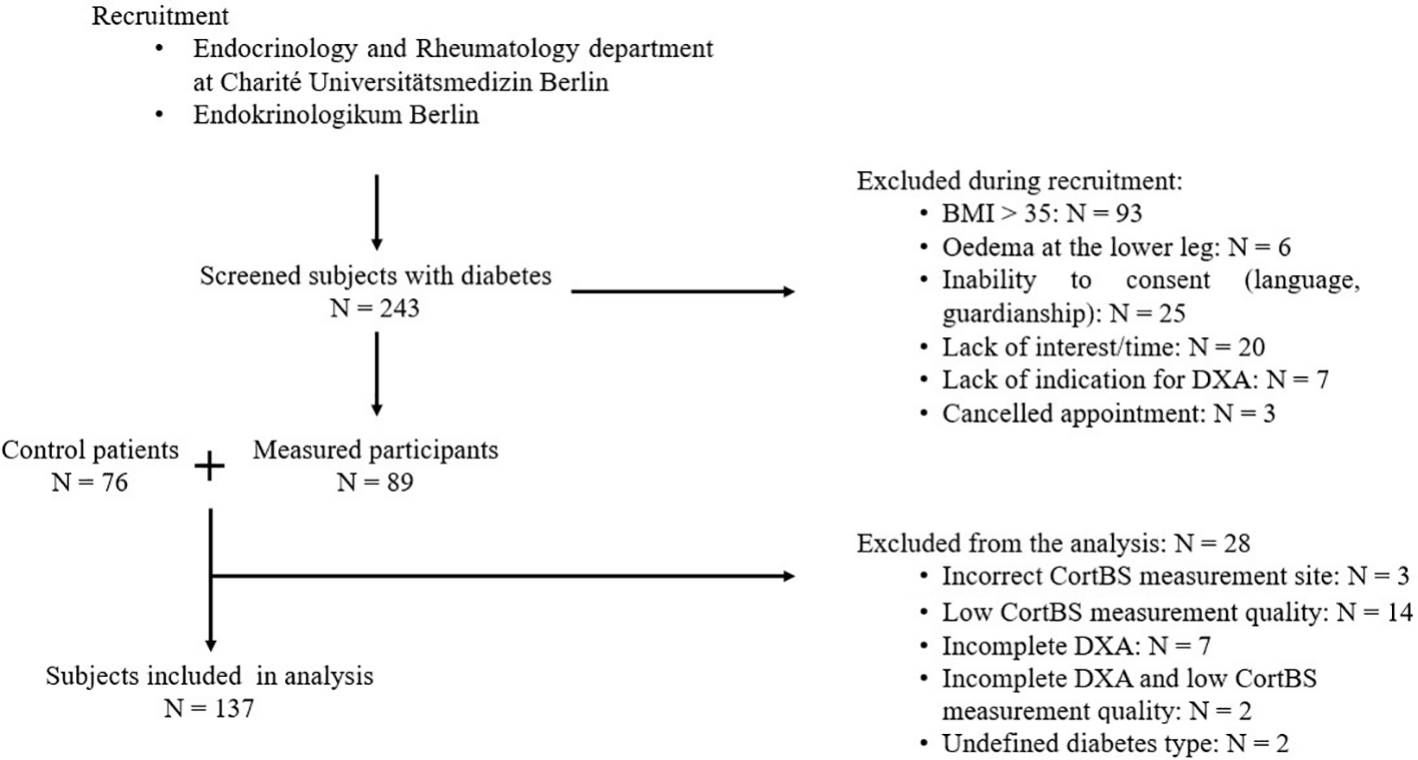
Flowchart of participant recruitment.

In this cross-sectional study, 89 men and women with diabetes mellitus of ages ranging from 49 to 81 were recruited between November 2022 to September 2023, as outlined in **Figure 1**. Patients at the endocrinological and rheumatological departments of the Charité Universitätsmedizin Berlin and a local doctor’s office specializing in diabetic patients (Endokrinologikum), were eligible for participation. Screened patients were excluded from further analysis if any of the following exclusion criteria were met: (1) BMI > 35 kg/m², (2) edema of the lower legs or metal implants in the lower legs, (3) inability to understand the nature of the study or inability to consent, (4) lack of indication for DXA scan or DXA scan older than one year.

During the measurement appointment, participants’ height and weight were measured, and a comprehensive medical history questionnaire was recorded. The assessment included details on diabetes type, disease duration, microvascular and macrovascular complications of diabetes, diabetes-specific medication, fracture history, lifestyle, and use of anti-osteoporotic drugs. Additionally, information on other medical conditions and medications was collected. Self-reported fractures from the past ten years, excluding fractures in fingers and toes, were considered eligible and verified with medical records when possible (40).Metabolic control in patients was assessed by measuring hemoglobin A1C (HbA1c) values at the time of the study visit or within a few days of the visit for some participants. Bone turnover parameters were assessed according to the German guidelines (40).

An age- and sex-matched control group of 76 non-diabetic patients, with and without fragility fractures, was formed from participants enrolled in a different study (“Identification of increased fracture risk through non-invasive determination of cortical micro- and macro-structural properties using quantitative bone ultrasound imaging” German Clinical Trial Register number: DRKS00025849). After recruitment, 26 patients were excluded from the analysis for the following reasons: CortBS measurement did not meet quality criteria (n = 14) or a bone surface was only detected in the anterior region (n = 3), incomplete DXA data, i.e., measurements could not be performed or analyzed at femur and spine (n = 7), CortBS quality criteria met and incomplete DXA data (n = 2), and undefined diabetes type other than T1DM or T2DM (n = 2). Diabetic participants were then stratified into four groups based on fracture history and diabetes type: (1) T2DM without fracture (T2DM^noFx^), (2) T2DM with fracture (T2DM^Fx^), (3) T1DM without fracture (T1DM^noFx^), and (4) T1DM with fracture (T1DM^Fx^).

All participants gave written consent before study participation. The study was registered in the German Clinical Trial Register (DRKS00029331) and approved by the local ethics committee of the Charité–University Hospital Berlin (EA4/140/22).

### DXA bone densitometry

DXA scans were performed using a medical narrow-angle fan beam densitometer, GE Lunar Prodigy, for the diabetes group and part of the control group, and GE Lunar iDXA for the remaining control patients, in accordance with the vendor’s manual. Seven participants from the diabetes group and two participants from the control group provided externally taken DXA scans (GE Lunar Prodigy device for diabetes patients) conducted less than one year prior to recruitment. We did not conduct a cross-calibration of the devices but consider the potential variations to have minimal impact on the results. For all other participants, measurements were taken both at the femora and the lumbar spine (L1-L4). Each lumbar spine scan was checked for artifacts to ensure that inadequate scans were excluded from the analysis. At least one femur scan and two valid vertebrae in the lumbar spine were necessary for a participant’s inclusion in the analysis. *aBMD* values and corresponding T-scores were assessed at the lumbar spine, femoral neck, and total proximal femur area. If measurements from both femoral sides were available, the lowest BMD and T-score values of the total area from either side were used for further analysis. If only one side was measured, the BMD and T-score values from that side were used.

### Quantitative bone ultrasound imaging

**Figure 2:**
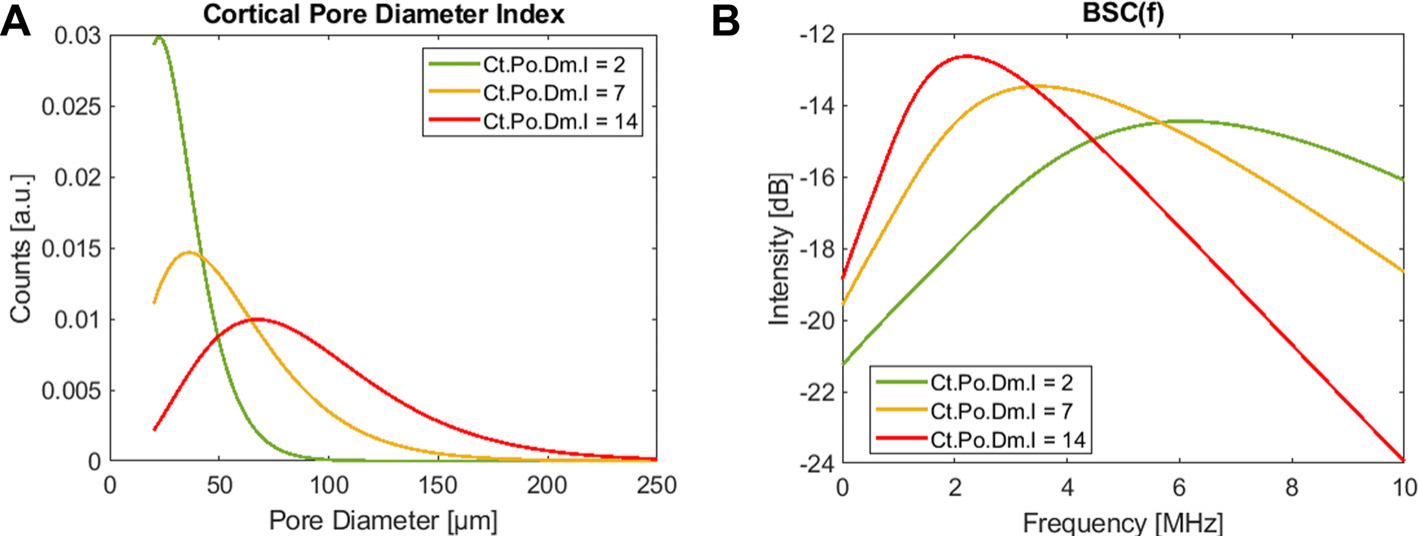
The intracortical pore diameter index *Ct.Po.Dm.I* is a monotonically increasing parameter that reflects the characteristic changes of the cortical pore diameter distribution **(A)** in response to imbalanced tissue remodeling. It is derived from fitting a corresponding theoretical backscatter coefficient **(B)** to the measured *BSC(f)*. The three representations resemble young healthy (green), a severe osteoporotic (yellow), and a “trabecularized” (red) cortical bone morphologies. Note that the amplitudes of *BSC(f)* and *Ct.PoDm.D* scale with pore density.

Ultrasound scans were performed with a medical ultrasound scanner, SonixTOUCH, equipped with a SonixDAQ single-channel data acquisition system and a 4DL14-5/38 3-D linear array transducer (Ultrasonix, Richmond, Canada). The system was operated via a custom-developed user interface to perform a Cortical Backscatter (CortBS) measurement, as described in (39). Briefly, the measurement consists of 1) an image-guided 3-D multidirectional scan of a slightly focused ultrasound beam across a selected cortical bone volume of interest, 2) the reconstruction of a 3D volume from the acquired channel data, 3) the automatic detection of the periosteal bone surface and the sound beam inclination for all acquisitions, 4) the calculation of an inclination-corrected reference spectrum from signals reflected from the periosteal bone interface, 5) the calculation of a normalized depth-dependent spectrogram, 6) the estimation of the frequency-dependent attenuation and backscatter coefficients *α(f)* and *BSC(f)*, respectively, and 7) the estimation of the intracortical pore diameter distribution *Ct.PoDm.D*. Subsequently, microstructural and viscoelastic parameters, the intracortical pore diameter index *Ct.Po.Dm.I* (see **Figure 2**) were derived. CortBS risk scores were calculated using multivariate logistic regression models based on ultrasound variables associated with prevalent fractures. Different scores were derived for vertebral, non-vertebral, and any type of fractures. For comparison with DXA T-scores, the means and standard deviations of the predictor variable were used. All variables obtained from the CortBS measurement are summarized in **Table 1**.

**Table 1:** Acoustic, microstructural, and viscoelastic parameters assessed by ultrasound.

Ultrasound Parameter	Meaning
α*_0_* [dB/mm]	The intercept value of a linear function fitted to *α(f)* is associated with the number and size of the intracortical pores, and tissue absorption in the sound propagation direction
α*_f_* [dB/mm/MHz]	The slope value of a linear function fitted to *α(f)* is associated with the size of the intracortical pores and viscoelastic tissue absorption in the sound propagation direction
α*_6-MHz_* [dB/mm]	The attenuation at the nominal center frequency (6 MHz) is an indicator of the apparent attenuation
*BSC_Mean_*	The mean amplitude of the measured backscatter coefficient reflects the intensity of scattered ultrasound waves and is influenced by the number and size of intracortical pores
*Ct.Po* [%]	The percentage of cortical porosity estimated using the pore diameter distribution and adjusted by BSC_Mean_ to account for variable pore density
*Ct.Po.Dm.D_peak_* [µm]	The peak of the pore diameter distribution provides insight on the most frequent pore diameter in the cortical bone
*Ct.Po.Dm.D_Q10_* [µm]	The 10^th^ percentile of the pore diameter distribution indicates the lower end of the distribution of pore sizes in the cortical bone
*Ct.Po.Dm.D_Q90_* [µm]	The 90^th^ percentile of the pore diameter distribution indicates the upper end of the distribution of pore sizes in the cortical bone
*Ct.Po.Dm.D_FWHM_* [µm]	The Full Width Half Maximum describes the variability of the pore size distribution around the most frequent pore size value
*Ct.Po.Dm.D_FWHM,min_* [µm]	The minimum crossing point of the FWHM values
*Ct.Po.Dm.D_FWHM,max_* [µm]	The maximum crossing point of the FWHM values
*AIB_Average_* [dB]	The average Apparent Integrated Backscatter amplitude is a common ultrasound parameter to assess cartilage and bone matrix degeneration
*Ct.Po.Dm.I*	The intracortical pore diameter index is a monotonically increasing parameter, which reflects changes in *Ct.Po.Dm.D* in response to unbalanced remodeling (**Figure 2**)
*CortBS Risk Score*	The CortBS risk scores are normalized prediction results of the PLS discrimination analysis, i.e., the model predictor variable was subtracted by the mean and divided by the standard deviation obtained from the non-fractured controls

All ultrasound measurements were taken at the central anteromedial tibia midshaft on the leg with the lowest femoral DXA T-Score. The tibia length was measured from the medial knee joint cleft to the medial malleolus, with both landmarks manually palpated and the 50% intersection marked with a skin marker pencil. Ultrasound gel and an ultrasound coupling pad (Aquaflex, Parker Laboratories, Inc., Fairfield, NJ, USA) were used to attach the transducer to the skin at the marked position. Conventional B-mode images facilitated the positioning of the probe, i.e. to center a cross-sectional image of the periosteal tibia bone interface. The probe was manually tilted until the bone surface was nearly perpendicular to the sound beam. Focus position was aligned with the periosteal bone interface (note that the software was programmed such that the focus was set 1 mm below to ensure that all beams are focused inside the cortical bone tissue), and gain was adjusted to obtain approximately 100% signal level at the periosteal bone interface. The multidirectional scan was performed automatically by means of 1) electronic beam steering and 2) sweeping the transducer array using the built-in motor in the direction perpendicular to the image plane.

Two measurements were taken for each participant to guarantee that at least one would meet the requisite quality standards. The data acquisition was performed within 3 seconds per scan. The signal quality of the captured channel data was inspected directly after completion of the scan. If the signal level was too low, the signal gain was readjusted, and the scan was repeated.

### Statistics

Continuous variables are presented as means and standard deviations (SD) or standard error (SE). Data were tested for normal distribution by a Lilliefors test. Differences with respect to sex, prevalent fractures, diabetes type, co-morbidities, and medications were analyzed using N-way ANOVA. If data were not normally distributed, observed effects were confirmed using non-parametric Wilcoxon rank sum tests. Associations between continuous variables were performed using stepwise multivariate regression analysis the calculation of Spearman’s rank sum correlation coefficient *ρ*.

The fragility fracture discrimination performance of CortBS and DXA was assessed by multivariate partial least squares (PLS) linear discrimination analysis with Leave-One-Out Cross-Validation (PLS-LDA LOOCV) using the libPLS library (41). For variable selection, a sub-window permutation analysis (SPA) using 2,000 Monte Carlo samplings was repeated until a stable set of significant model variables was found. The number of PLS components was restricted to one-tenth of the observation number. Different discrimination models were developed to predict vertebral, non-vertebral, and any fragility fractures from DXA-based BMD values and T-scores, CortBS parameters, and for combinations of DXA or CortBS parameters with each participant’s anthropometric data (weight, height, BMI) and age. The mean and SE of the area under the curve (AUC) of the receiver operation characteristics (ROC), accuracy, and sensitivity were calculated. Differences between the AUC values were evaluated using the Hanley & McNeil test using MedCalc 22.032 (MedCalc Software Ltd, Ostend, Belgium).

To evaluate the correlation of multiple QUS parameters with DXA BMD, PLS regression with threefold cross-validation was used. Spearman’s rank sum correlation coefficient ρ and root mean square error (RMSE) between the predicted QUS-based parameter and those measured by DXA were computed.

Except for the PLS-LOOC and SPA analyses, all statistical tests were performed using the Statistics Toolbox of Matlab R2023b (MathWorks, Natick, MA, USA). Statistical results were considered significant for *p* values < 0.05.

## Results

### Study population

During the recruitment phase, the diabetic group initially included 39 T1DM, 48 T2DM patients, and 2 patients with undefined diabetes type. However, due to the exclusion criteria, data from 31 T1DM and 35 T2DM patients were finally used in the statistical analysis. Among the T1DM patients, eight had at least one fracture. Of those, one had a vertebral fracture, seven had non-vertebral fractures, and none had both types of fractures. Among the T2DM patients, 17 patients had at least one fracture. Of those, four had vertebral fractures, 9 had a non-vertebral fracture, and four had both. Three patients with T2DM and a previous fracture received antiresorptive treatment. Vitamin D intake was observed in 42 patients of the diabetes cohort.

In the analyzed diabetes population, 32 subjects were females, all of whom were postmenopausal. The average duration of diabetes was 19 ± 15 years. Forty-five subjects were on insulin, and 29 were on oral antidiabetic drug treatment, with metformin being the most common (n = 24). No patients were on thiazolidines, sulfonylureas or glucosidase inhibitors at the time of the study. The average HbA1c value of diabetic patients was 7.2 ± 1.2. Eleven patients in the T1DM group were diagnosed with late-onset autoimmune diabetes in adults (LADA).

Further patient characteristics in juxtaposition to controls are summarized in **Table 2**. The patient’s characteristics were similar in the control and diabetes groups, except for the expected differences in anthropometric data and slightly higher mean age. No significant differences were observed between fractured and non-fractured groups in the control, T1DM, and T2DM groups regarding anthropomorphic data and medical history.

**Table 2:** Age, anthropometric data (range, means, and standard deviations), disease, and medication history of controls and patients with T1DM and T2DM with (Fx) and without (nFx) fragility fractures.

Parameter	All patients				Control				T1DM				T2DM			
	Range	All (n=137)	Fx (n=41)	nFx (n=96)	Range	All (n=71)	Fx (n=16)	nFx (n=55)	Range	All (n=31)	Fx (n=8)	nFx (n=23)	Range	All (n=35)	Fx (n=17)	nFx (n=18)
**Age (years)**	49.00 - 79.00	66.29 ± 7.00	67.13 ± 6.55	65.90 ± 7.20^d^	60.00 - 79.00	69.11 ± 5.40	69.17 ± 3.52	69.09 ± 5.91	51.00 - 79.00	62.32 ± 7.83	66.00 ± 9.23	61.04 ± 7.06	49.00 - 77.00	64.54 ± 6.64	65.75 ± 7.26	63.26 ± 5.85
**Height (cm)**	151.00 - 192.00	167.94 ± 9.14	168.28 ± 8.07	167.78 ± 9.62^s, d^	152.00 - 183.00	164.41 ± 7.12	166.78 ± 6.87	163.64 ± 7.10	152.00 - 192.00	173.55 ± 10.26	169.62 ± 11.10	174.91 ± 9.84	151.00 - 186.00	169.69 ± 8.93	169.10 ± 7.93	170.32 ± 10.05
**Weight (kg)**	44.00 - 122.00	72.93 ± 16.69	75.22 ± 16.87	71.88 ± 16.59^s, d^	44.00 - 88.00	64.88 ± 10.54	68.33 ± 10.37	63.77 ± 10.45	50.00 - 122.00	78.00 ± 18.26	72.62 ± 20.01	79.87 ± 17.70	50.00 - 114.00	83.77 ± 17.50	82.45 ± 18.09	85.16 ± 17.22
**Body mass index (kg/m^2^)**	16.56 - 36.03	25.67 ± 4.40	26.45 ± 5.01	25.31 ± 4.07^d, s^	16.56 - 31.18	23.98 ± 3.44	24.61 ± 3.78	23.78 ± 3.33	18.82 - 36.03	25.64 ± 4.23	24.95 ± 5.11	25.88 ± 3.99	17.30 - 35.83	28.90 ± 4.51	28.70 ± 5.25	29.11 ± 3.70
**Sex (M / F)**		47 / 90	15 / 26	32 / 64		13 / 58	6 / 10	7 / 48		18 / 13	2 / 6	16 / 7		16 / 19	7 / 10	9 / 9
Diseases																
** Diabetes**		66	25	41		0	0	0		31	8	23		35	17	18
** Rheumatic diseases**		31	7	24		27	6	21		1	0	1		3	1	2
** Other chronic inflammatory diseases**		26	7	19		18	3	15		2	1	1		6	3	3
** Hyperthyreosis**		2	0	2		2	0	2		0	0	0		0	0	0
Medication																
** Antiresorptive**		29	13	16		26	10	16		0	0	0		3	3	0
** Anabolic treatment**		6	2	4		6	2	4		0	0	0		0	0	0
** Vitamin D**		104	30	74		62	15	47		18	4	14		24	11	13
** Corticoids**		9	5	4		0	0	0		3	1	2		6	4	2
** Aromatase inhibitors**		1	0	1		1	0	1		0	0	0		0	0	0
** Other medications**		86	26	60		57	11	46		13	6	7		16	9	7

s, Sex (Male, Female); d, Diabetes type (Control, T1DM, T2DM); f, Fracture (Fx, nFx).

Significant differences are marked in bold.

### Bone mineral density is reduced in patients with fractures but higher in patients with T2DM

Complete spine and femur measurements were missing for 5 and 4 patients, respectively, leading to their exclusion from subsequent analysis. A strong positive association with weight or BMI was observed for all DXA parameters (**Supplementary Table A1**). Height had a minor negative association with the spine and total femur *aBMD*. All *aBMD* values and T-scores were significantly reduced in patients who were treated with antiresorptive or anabolic drugs, with the reduction more pronounced in femur measurements compared to spine measurements. Minor associations with other drug treatments were also observed. Differences with respect to fractures and DM type are summarized in **Table 3** and **Supplementary Table A1**. DXA *aBMD* values were significantly reduced in patients with fractures, particularly in total femur T scores (F = 11.3) and to a lesser extent in the spine T scores (F = 4.7). After adjustment for the impact of fractures, all T scores and *aBMD* values were significantly higher in patients with T2DM compared to controls (4.7 ≤ F ≤ 15.1). The T scores and *aBMD* values in patients with T1DM fell between those observed in controls and patients with T2DM, with some significant differences compared to controls or T2DM. It should be noted that the relative impact of DM on the DXA *aBMD* values in the spine or total femur was much stronger than that of prevalent fractures. Moreover, the mean T scores for all groups with fractures were higher than -2.5, thus not meeting the criteria for an osteoporotic T-Score.

**Table 3:** DXA parameters (range, means, and standard deviations) of patient cohorts with (Fx) and without (nFx) any fractures.

Parameter	All patients				Control				T1DM				T2DM			
	Range	All (n=137)	Fx (n=41)	nFx (n=96)	Range	All (n=71)	Fx (n=16)	nFx (n=55)	Range	All (n=31)	Fx (n=8)	nFx (n=23)	Range	All (n=35)	Fx (n=17)	nFx (n=18)
**aBMD_Femur(Neck)_ (g/cm^2^)**	0.42 - 1.26	0.83 ± 0.15	**0.78 ± 0.16**	**0.85 ± 0.14 ^d^**	0.56 - 1.10	0.79 ± 0.11	0.74 ± 0.09	0.80 ± 0.11	0.52 - 1.15	0.83 ± 0.14	0.77 ± 0.13	0.86 ± 0.15	0.42 - 1.26	0.90 ± 0.19	0.83 ± 0.21	0.96 ± 0.16
**aBMD_Femur(Total)_ (g/cm^2^)**	0.47 - 1.44	0.87 ± 0.17	**0.83 ± 0.19**	**0.89 ± 0.16 ^d^**	0.56 - 1.16	0.81 ± 0.12	0.75 ± 0.09	0.83 ± 0.13	0.49 - 1.28	0.89 ± 0.16	0.82 ± 0.17	0.91 ± 0.16	0.47 - 1.44	0.97 ± 0.22	0.90 ± 0.24	1.03 ± 0.19
**aBMD_Spine_ (g/cm^2^)**	0.52 - 1.60	1.07 ± 0.20	**1.04 ± 0.22**	**1.08 ± 0.19 ^d^**	0.58 - 1.43	1.01 ± 0.18	0.93 ± 0.11	1.03 ± 0.19	0.72 - 1.46	1.11 ± 0.17	1.14 ± 0.23	1.10 ± 0.16	0.52 - 1.60	1.16 ± 0.23	1.10 ± 0.26	1.22 ± 0.17
**T-score_Femur(Neck)_**	-5.00 - 3.60	-1.41 ± 1.23	**-1.65 ± 1.51**	**-1.31 ± 1.09^d^**	-3.50 - 3.60	-1.59 ± 1.10	-1.78 ± 1.66	-1.53 ± 0.89	-3.80 - 0.60	-1.53 ± 1.19	-1.90 ± 1.10	-1.40 ± 1.21	-5.00 - 1.50	-0.96 ± 1.43	-1.40 ± 1.57	-0.54 ± 1.17
**T-score_Femur(Total)_**	-4.80 - 2.70	-1.31 ± 1.34	**-1.67 ± 1.45**	**-1.15 ± 1.26 ^d^**	-3.80 - 1.40	-1.68 ± 1.01	-2.31 ± 0.73	-1.50 ± 1.01	-4.20 - 1.50	-1.25 ± 1.35	-1.62 ± 1.42	-1.13 ± 1.34	-4.80 - 2.70	-0.60 ± 1.62	-1.09 ± 1.77	-0.13 ± 1.35
**T-score_Spine_**	-5.70 - 3.20	-0.98 ± 1.64	**-1.22 ± 1.87**	**-0.88 ± 1.53 ^d^**	-4.90 - 1.90	-1.50 ± 1.46	-2.29 ± 1.00	-1.27 ± 1.50	-4.10 - 2.40	-0.69 ± 1.47	-0.41 ± 1.88	-0.79 ± 1.34	-5.70 - 3.20	-0.19 ± 1.77	-0.59 ± 2.09	0.19 ± 1.37

Significant differences are marked in bold.

s, Sex; d, Diabetes type; f, Fracture.

Significant differences obtained from multivariate ANOVA with respect to any prevalent fractures are marked in bold; additional associations with respect to diabetes are marked by the superscript letter ‘d’. Additional information about differences with respect to diabetes type, age, sex, anthropometric data, co-morbidities, and medication is provided in the **Supplementary Table A1**.

### CortBS reveals distinct differences in cortical pore morphology associated with fractures and diabetes

The measurements of 3 patients were excluded, because only the anterior, but not the antero-medial region of the tibia was visible in the image. Moreover, the measurements of 16 patients (of which 2 had incomplete DXA), had to be discarded due to low quality.

Most anthropometric parameters were not associated with CortBS parameters. The mean attenuation and backscatter amplitudes (α_6MHz_ and BSC_mean_) were significantly reduced in participants with higher weight and patients with T2DM (**Supplementary Table A1**). Moreover, height had a minor negative association with α_0_. Several parameters, particularly α_0_ (F = 19.9), were affected by sex. Antiresorptive and anabolic drug treatments had distinct effects on different CortBS parameters. While pore diameter distributions did not significantly differ in patients treated with antiresorptive drugs, they were increased in those treated with anabolic drugs. Conversely, backscatter and attenuation coefficients changed in patients on antiresorptive drugs. Porosity was increased in patients receiving either treatment. Differences related to fractures and DM are summarized in **Table 4**. All CortBS parameters, except for α_6MHz_ and BSC_mean_, were significantly altered in patients with fractures. The most pronounced association was observed in the CortBS risk score (F = 15.7) and, to the least extent, in cortical porosity (F = 5.1). Notably, only the parameters that were not associated with fractures (α_6MHz_ and BSC_mean_) were significantly reduced in patients with T2DM compared to controls (4.2 ≤ F ≤ 7.3).

**Table 4:** CortBS parameters (range, means, and standard deviations) of patient cohorts with (Fx) and without (nFx) fractures.

Parameter	All patients				Control				T1DM				T2DM			
	Range	All (n=137)	Fx (n=41)	nFx (n=96)	Range	All (n=71)	Fx (n=16)	nFx (n=55)	Range	All (n=31)	Fx (n=8)	nFx (n=23)	Range	All (n=35)	Fx (n=17)	nFx (n=18)
**Ct.α_o_(dB/mm)**	0.38 - 2.69	1.68 ± 0.50	**1.81 ± 0.42**	**1.63 ± 0.52^s, s:f^**	0.97 - 2.69	1.81 ± 0.45	1.91 ± 0.38	1.78 ± 0.47	0.42 - 2.41	1.55 ± 0.54	1.78 ± 0.44	1.48 ± 0.56	0.38 - 2.44	1.54 ± 0.49	1.72 ± 0.46	1.36 ± 0.46
**Ct.α_f_(dB/MHz/mm)**	-0.07 - 0.30	0.10 ± 0.08	**0.07 ± 0.07**	**0.11 ± 0.07^s^**	-0.07 - 0.26	0.09 ± 0.07	0.05 ± 0.06	0.10 ± 0.07	0.00 - 0.30	0.12 ± 0.08	0.12 ± 0.06	0.13 ± 0.08	-0.07 - 0.24	0.10 ± 0.08	0.06 ± 0.07	0.13 ± 0.07
**Ct.α_6MHz_(dB/mm)**	1.34 - 3.08	2.27 ± 0.30	2.20 ± 0.28	2.30 ± 0.31^s, d^	1.72 - 3.08	2.35 ± 0.30	2.21 ± 0.29	2.39 ± 0.30	1.81 - 2.71	2.28 ± 0.24	2.45 ± 0.17	2.22 ± 0.23	1.34 - 2.54	2.09 ± 0.29	2.08 ± 0.23	2.11 ± 0.35
**Ct.Po(%)**	2.12 - 42.57	8.89 ± 7.22	**10.99 ± 9.00**	**7.99 ± 6.15**	2.76 - 42.57	9.44 ± 7.54	11.65 ± 9.41	8.80 ± 6.87	2.18 - 27.78	7.19 ± 5.23	7.36 ± 2.76	7.13 ± 5.90	2.12 - 41.41	9.26 ± 8.00	12.08 ± 10.39	6.59 ± 3.28^s^
**Ct.Po.Dm._DPeak_(μm)**	20.00 - 56.00	28.94 ± 6.07	**31.64 ± 6.88**	**27.78 ± 5.32**	20.00 - 56.00	28.61 ± 6.02	**31.75 ± 7.46**	**27.69 ± 5.27**	20.00 - 39.00	28.23 ± 5.54	28.38 ± 3.89	28.17 ± 6.09	21.00 - 48.67	30.24 ± 6.57	**33.08 ± 7.20**	**27.56 ± 4.67**
**Ct.Po.Dm._DQ10_(μm)**	20.00 - 34.00	22.58 ± 2.26	**23.54 ± 2.80**	**22.17 ± 1.86**	20.00 - 34.00	22.44 ± 2.26	**23.56 ± 3.12**	**22.11 ± 1.85**	20.00 - 26.00	22.39 ± 1.89	22.25 ± 1.16	22.43 ± 2.11	20.00 - 30.67	23.04 ± 2.55	**24.14 ± 2.95**	**22.00 ± 1.57**
**Ct.Po.Dm._DQ90_(μm)**	46.00 - 139.00	70.20 ± 19.10	**77.60 ± 21.68**	**67.05 ± 17.04**	46.00 - 139.00	68.87 ± 18.38	**77.12 ± 21.01**	**66.47 ± 17.02**	46.00 - 110.00	68.66 ± 18.17	66.38 ± 10.81	69.45 ± 20.27	49.00 - 132.00	74.28 ± 21.17	**83.33 ± 24.75**	**65.72 ± 12.68**
**Ct.Po.Dm._DFWHM_(μm)**	14.60 - 84.70	35.47 ± 14.29	**41.25 ± 15.72**	**33.01 ± 12.96**	14.60 - 84.70	34.51 ± 13.75	**40.92 ± 15.11**	**32.64 ± 12.88**	14.60 - 62.90	34.04 ± 13.85	33.06 ± 8.88	34.38 ± 15.36	18.00 - 80.10	38.70 ± 15.62	**45.42 ± 17.80**	**32.36 ± 10.10^s^**
**Ct.Po.Dm._DFWHM,min_(μm)**	20.00 - 25.70	20.05 ± 0.51	20.18 ± 0.92	20.00 ± 0.00	20.00 - 25.70	20.08 ± 0.68	20.36 ± 1.42	20.00 ± 0.00^s^	20.00 - 20.00	20.00 ± 0.00	20.00 ± 0.00	20.00 ± 0.00	20.00 - 21.67	20.05 ± 0.28	20.10 ± 0.40	20.00 ± 0.00
**Ct.Po.Dm._DFWHM,max_(μm)**	34.60 - 110.40	55.53 ± 14.48	**61.43 ± 16.22**	**53.01 ± 12.96**	34.60 - 110.40	54.59 ± 14.06	**61.28 ± 16.24**	**52.64 ± 12.88**	34.60 - 82.90	54.04 ± 13.85	53.06 ± 8.88	54.38 ± 15.36	38.00 - 100.10	58.75 ± 15.74	**65.52 ± 17.98**	**52.36 ± 10.10^s^**
**Ct.AIB_Average_**	-8.15 - -0.65	-1.80 ± 0.88	-1.67 ± 0.75	-1.86 ± 0.93^s:d^	-3.97 - -0.65	-1.67 ± 0.60	-1.74 ± 0.82	-1.65 ± 0.52	-5.37 - -0.92	-2.04 ± 0.84	-1.60 ± 0.62	-2.20 ± 0.87^s^	-8.15 - -0.81	-1.86 ± 1.29	-1.63 ± 0.78	-2.08 ± 1.63
**Ct.BSC_Mean_**	-24.89 - -7.88	-14.65 ± 2.34	-14.60 ± 2.21	-14.67 ± 2.41^s, d^	-18.40 - -7.88	-13.95 ± 2.18	-13.88 ± 2.47	-13.97 ± 2.11	-19.77 - -10.90	-15.19 ± 1.98	-14.21 ± 1.59	-15.53 ± 2.02	-24.89 - -10.92	-15.58 ± 2.55	-15.47 ± 1.99	-15.68 ± 3.05
**Ct.Po.Dm.I**	1.00 - 12.00	3.96 ± 2.07	**4.85 ± 2.39**	**3.58 ± 1.80**	1.00 - 12.00	3.89 ± 2.05	**4.81 ± 2.41**	**3.63 ± 1.87**	1.00 - 8.00	3.51 ± 1.79	3.69 ± 1.28	3.45 ± 1.96	1.50 - 10.50	4.48 ± 2.30	**5.43 ± 2.67**	**3.58 ± 1.46**

Significant differences are marked in bold.

s, Sex; d, Diabetes; f, Fracture.

Significant differences obtained from multivariate ANOVA with respect to any prevalent fractures are marked in bold; additional associations with respect to sex, diabetes, and coupled effects with fractures are marked by the superscript letters ‘s’, ‘d’, and ‘:’, respectively. Additional information about differences with respect to diabetes type, age, sex, anthropometric data, co-morbidities, and medication is provided in the **Supplementary Table A1**.

**Figure 3:**
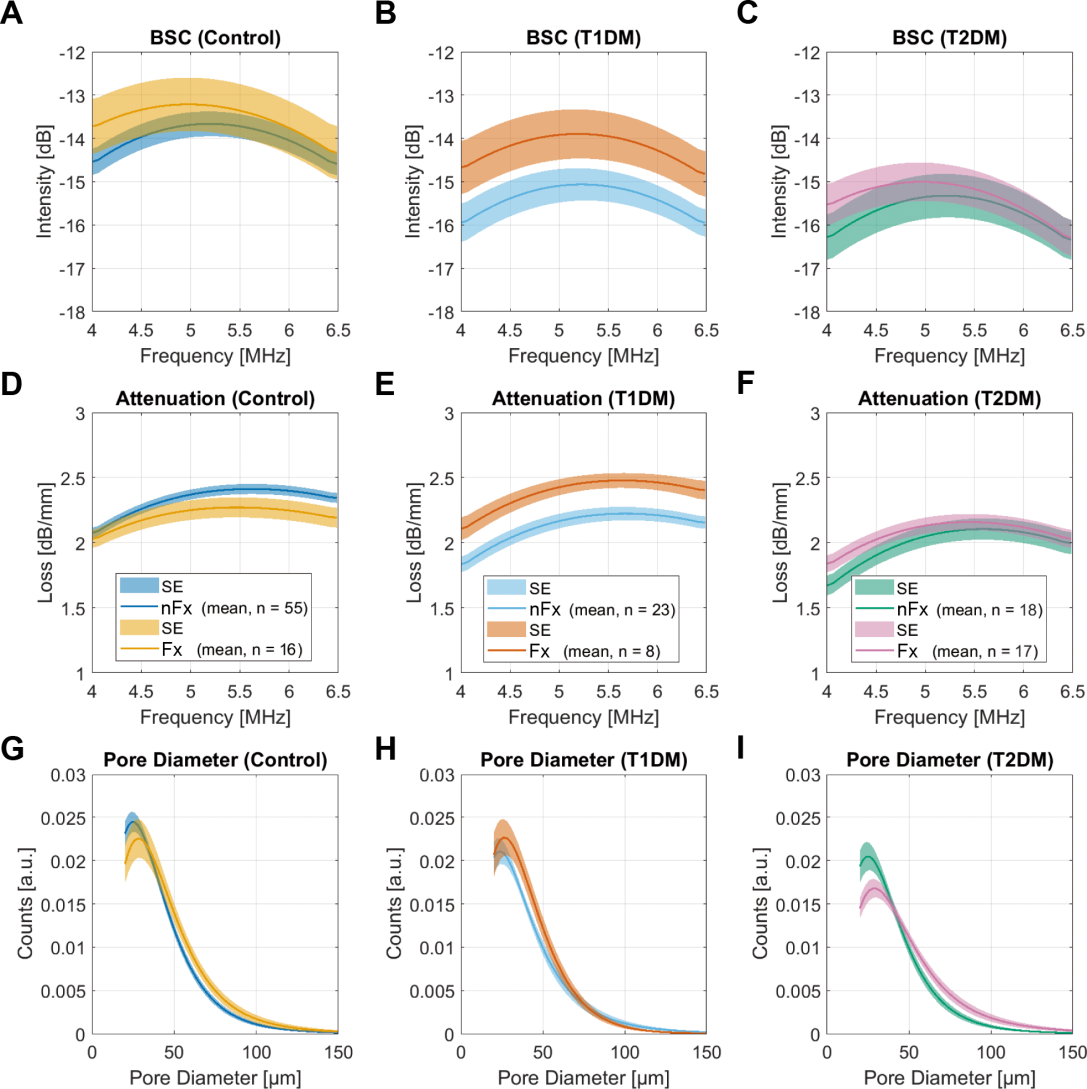
Backscatter **(A-C)** and attenuation **(D–F)** coefficients, and pore diameter distributions **(G–I)**, and pore diameter distributions in the control group and in patients with T1DM and T2DM. The solid lines and shaded areas indicate the means and standard errors of all participants in the respective groups. Note that the amplitudes of the pore diameter distributions **(G–I)** are scaled to the total porosity.

The mean backscatter and attenuation coefficients and pore diameter distributions in control and diabetes groups are shown in **Figure 3**. In the control group, individuals with fractures exhibited a shift in BSC peak towards smaller frequencies (**Figure 3A**), which was associated with an altered pore diameter distribution (**Figure 3G**). In particular, the number of small pores was reduced, the peak position was shifted towards larger values, and the number of large pores was increased. This was reflected in significant increases in pore size distribution parameters, e.g., peak, 10% and 90% quantiles, FWHM, FWHM_max_, pore diameter distribution index, and cortical porosity (**Table 4**, **Supplementary Table A1**). Moreover, the frequency-dependent attenuation was significantly reduced (**Table 4**, **Figure 3B**). Similar differences were observed between individuals with or without fractures in the T2DM group (**Table 4**, **Figures 3C, F, I**). However, backscatter and attenuation amplitudes were lower compared to the control group. This difference was significant, as quantified by *BSC_mean_* and α_6MHz_ (**Supplementary Table A1**), indicating that the scatterer density (i.e., pore density) was lower in T2DM patients compared to controls. Similar to the controls, prevalent fractures in T2DM patients were associated with the accumulation of large pores. In contrast, the T1DM group exhibited distinct differences compared to both the control and T2DM groups (**Figures 3B, E, H**). BSC and attenuation in patients with diabetes without fractures had a similar shape but were lower in amplitude compared to controls. This difference was reflected in significantly reduced *α_6MHz_* (F = 7.3) and BSC_mean_ (F = 4.2) values (**Table 4**, **Supplementary Table A1**). Notably, the pore diameter distribution in T1DM patients with fractures was similar to that of controls without fractures. In these patients, backscatter and attenuation coefficients matched the shape and amplitude of those in controls. These results suggest that T1DM patients without fractures had a lower pore density compared to healthy controls. In contrast, T1DM subjects with fractures exhibited a pore diameter distribution comparable to that of controls without fractures, indicating that pore density and diameter distribution in T1DM patients with fractures reach the level of controls without fractures.

**Figure 4:**
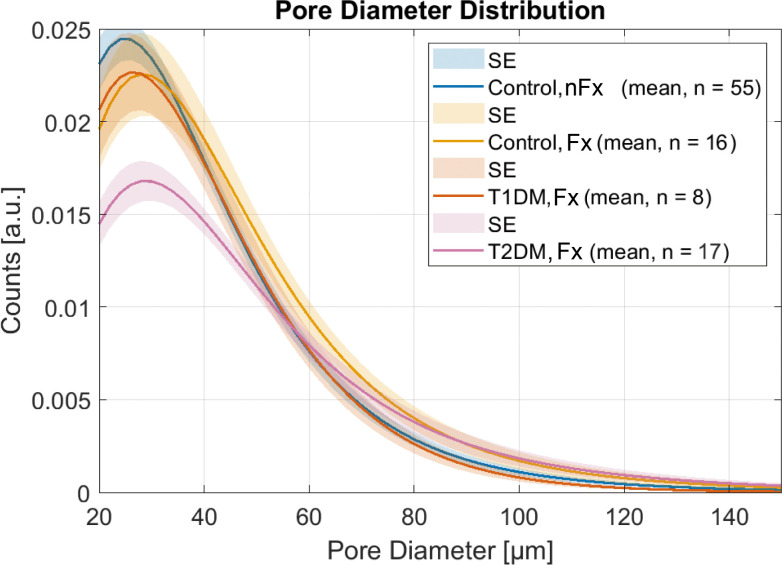
Pore diameter distributions in patients with fractures compared to healthy controls show a strong reduction of small pores and the highest number of large pores in the T2DM group, while the T1DM group with fractures has a pore diameter distribution similar to controls without fractures. Note that the amplitudes of the pore diameter distributions are scaled to the total porosity.

The distinct differences in pore diameter distribution with respect to fractures and diabetes type compared to controls without fractures are shown in **Figure 4**. The T2DM group exhibited the strongest reduction in normal-sized pores and the highest increase in large pores. In contrast, T1DM patients with fractures exhibited similar pore morphology to controls without fractures, with the exception of a non-significant reduction in the number of small pores.

### CortBS shows improved discrimination of prevalent fractures compared to DXA

**Figure 5:**
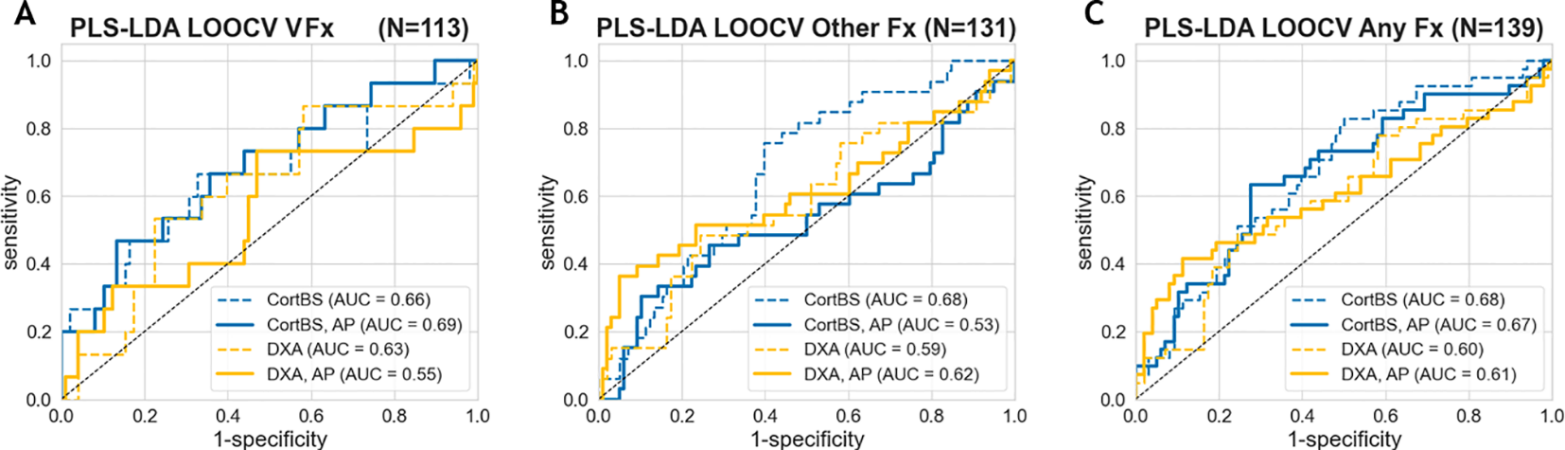
Fracture discrimination performance of CortBS and DXA for vertebral fractures **(A)**, other fractures **(B)**, and all fractures **(C)**. Dashed lines indicate the results from the best DXA and CortBS Risk Score only, and solid lines show the results with the incorporation of anthropometric (AP) parameters, including age, weight, height, and BMI, using the PLS discrimination models.

**Figure 5** shows the results of the discrimination analyses based on prevalent fractures. The AUC values obtained from the CortBS discrimination models (0.66 ≤ AUC ≤ 0.68) were significantly higher than those from DXA (0.59 ≤ AUC ≤ 0.63, best models from either BMD values or T-scores, see **Table 5**) for each type of fractures, reaching a significance level of p < 0.0001 for all fracture types. The combination of age and anthropometric information in the models resulted in a marginal improvement in the CortBS discrimination model for vertebral fractures (from AUC = 0.66 to AUC = 0.69) and in the DXA discrimination for other fractures (from AUC = 0.59 to AUC = 0.61). In other models, the additional degrees of freedom introduced by a higher number of variables rather led to a reduction in the discrimination performance.

**Table 5:** Fracture discrimination performance.

Model	Sensitivity	Specificity	AUC (SE)	Accuracy	Variables
Vertebral Fractures (Fx/nFx)					
** DXA (15/98)**	0.67	0.49	0.63 (<0.001)	0.51	*T-score.Femur(Total)*
** DXA + AP (15/98)**	0.73	0.53	0.55 (0.04)	0.56	*T-score.Femur(Total)* *T-score.Femur(Neck)* Weight Height BMI
** CortBS (15/98)**	0.67	0.62	0.66 (<0.001)	0.63	*Ct.α_6-MHz_* *Ct.Po.Dm.D_peak_* *Ct.Po.Dm.D_90_* *Ct.Po.Dm.I* *Ct.Po*
** CortBS + AP (15/98)**	0.67	0.58	0.69 (0.001)	0.59	*Ct.α_6-MHz_* *Ct.Po.Dm.D_mean_* *Ct.Po.Dm.D_FWHMmax_* *Ct.AIB_Average_* *Ct.Po.Dm.D_peak_*
Other Fractures (Fx/nFx)					
** DXA (33/98)**	0.63	0.49	0.59 (<0.001)	0.53	*T-score.Femur(Total)*
** DXA + AP (33/98)**	0.58	0.54	0.61 (0.04)	0.55	*T-score.Femur(Total)* *T-score.Spine* *Weight* *Age* *BMI*
** CortBS (33/98)**	0.76	0.60	0.68 (<0.001)	0.64	*Ct.α_6-MHz_* *Ct.Po.Dm.D_peak_* *Ct.Po* *Ct.BSC_Mean_* *Ct.AIB_Average_*
** CortBS + AP (33/98)**	0.48	0.56	0.53 (<0.001)	0.54	*Ct.BSC_Mean_* *Ct.AIB_Average_* Weight Height BMI
All Fractures (Fx/nFx)					
** DXA (41/98)**	0.66	0.49	0.60 (<0.001)	0.54	*T-score.Femur(Total)*
** DXA + AP (41/98)**	0.60	0.51	0.61 (0.04)	0.54	*T-score.Femur(Total)* *T-score.Spine* Weight Age BMI
** CortBS (41/98)**	0.66	0.60	0.68 (<0.001)	0.62	*Ct.α_6-MHz_* *Ct.Po.Dm.I* *Ct.Po.Dm.D_peak_* *Ct.AIB_Average_* *Ct.BSC_Mean_*
** CortBS + AP (41/98)**	0.66	0.61	0.67 (<0.001)	0.63	*Ct.Po.Dm.D_peak_* *Ct.AIB_Average_* *Ct.α_6-MHz_* Weight Height

PLS-LOOC discrimination models were created for each individual measurement modality separately and in combination with anthropometric (AP) data, age, and sex. The variables used for the models are summarized in the last column. Class proportions are shown as Fx (patients with fractures) and nFx (patients without fractures). The values in parenthesis in the first column indicate the number of fractured (Fx) and non-fractured (nFx) cases.

Bold values in the first column emphasize the measurement modalities and additional information such as anthropometric (AP) data, age, and sex used in the discrimination models.

Among the evaluated DXA parameters, the total femur T-score provided the best discrimination performance for all fracture types (**Table 5**). Distinct combinations of CortBS parameters were selected to discriminate different fracture types.

### Impacts of diabetes and fracture types on DXA and CortBS scores

**Figure 6:**
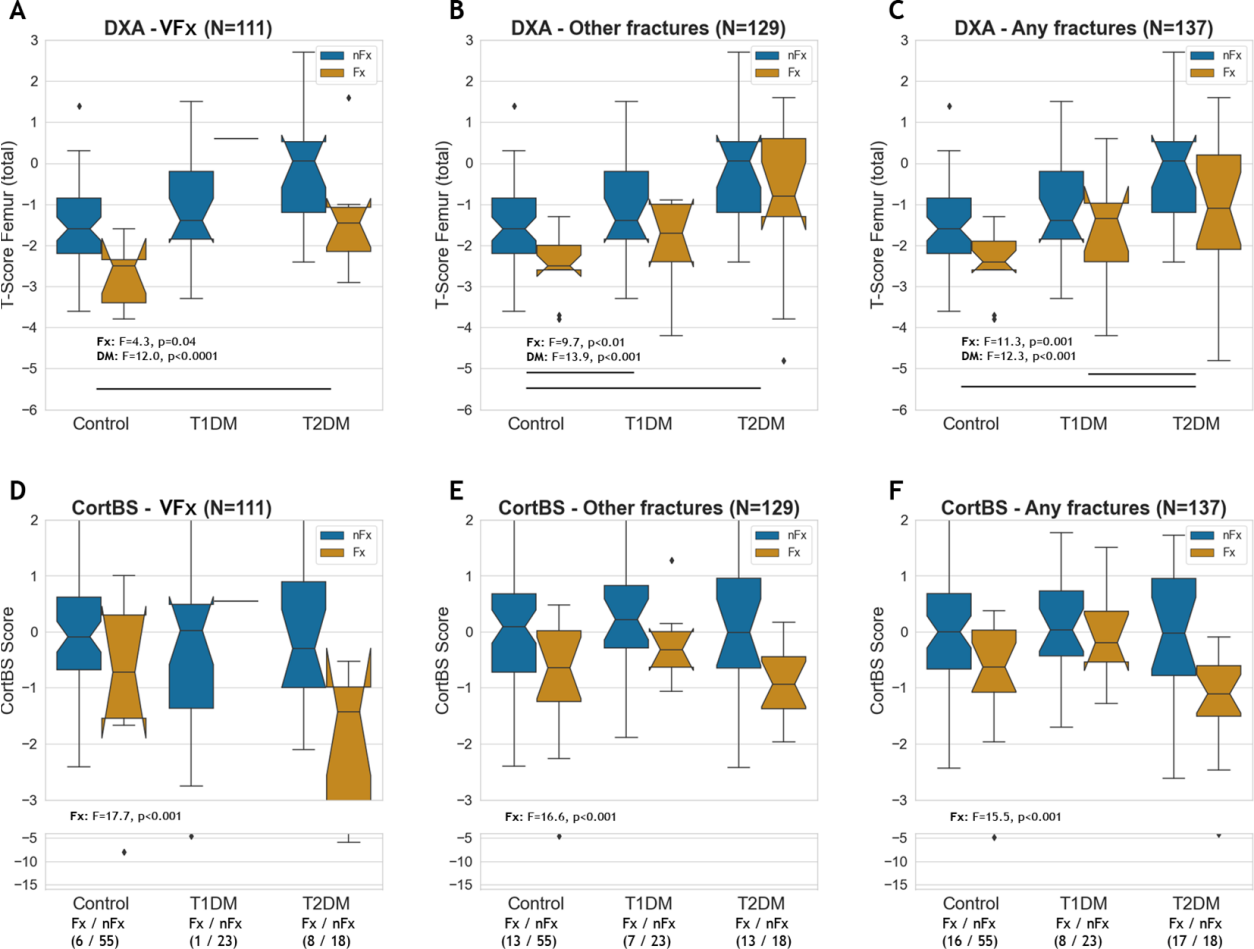
DXA total femur T-score **(A–C)** and CortBS risk score **(D–F)** in controls, T1DM and T2DM patients with and without vertebral, other and any type of fractures.

The total femur T-scores were significantly reduced in patients with fractures, but they were also affected by diabetes and diabetes type (**Figures 6A–C**). Notably, the impact of diabetes (12.0 ≤ F ≤ 13.9) was considerably greater than that of prevalent fractures (4.3 ≤ F ≤ 11.3). The CortBS risk scores were significantly reduced in patients with fractures across all fracture types (**Figures 6D–F**) and notably, were not significantly affected by diabetes.

### Associations of ultrasound parameters with bone mineral density

**Figure 7:**
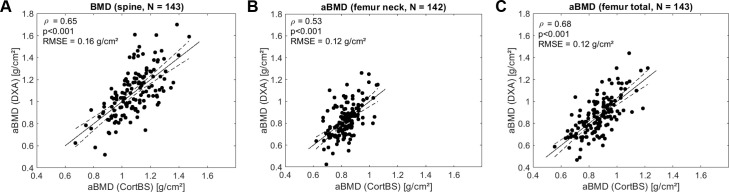
Predictions of *aBMD* at spine **(A)**, femur neck **(B)**, and total femur **(C)** based on ultrasound backscatter and anthropometric parameters using multivariate PLS regression Spearman correlation. RMSE, is the root mean squared error.

The prediction of *aBMD* from CortBS parameters using multivariate PLS models is shown in **Figure 7**. Moderate correlations were observed for all prediction models (0.53 ≤ ρ ≤ 0.68).

## Discussion

In this study, intracortical ultrasound backscatter measurements were conducted on patients with T1DM, T2DM, and age- and sex-matched non-diabetic controls. Patients with both types of diabetes exhibited decreased levels of backscatter and attenuation amplitudes. Significant associations were found between alterations in intracortical microstructure and the prevalence of both vertebral and non-vertebral fractures, regardless of the type and prevalence of diabetes. Notably, T1DM patients with fractures showed a different intracortical pore morphology compared to both controls and T2DM patients with fractures. To our knowledge, this is the first study to quantify intracortical pore diameter morphologies down to 20 µm in DM patients. The use of CortBS seems to improve discrimination performance compared to that of DXA. The association between CortBS parameters with *aBMD* was found to be significant. However, the moderate correlation coefficients support the hypothesis that the CortBS measurement is sensitive to structural and viscoelastic cortical bone tissue alterations. These alterations are not detectable by DXA and CortBS might provide a better reflection of the underlying bone pathology in both diabetes types.

The currently established diagnostic technologies have limited ability to evaluate the various structural and tissue matrix changes in bone caused by diabetes mellitus. The widely used DXA-based *aBMD* underestimates fracture risk when compared to non-diabetic individuals. The mechanisms underlying this risk underestimation at normal or even increased levels of *aBMD* are poorly understood. Nonetheless, fracture risk has been shown to be influenced by factors, such as diabetes type, fracture location, disease duration, and glycemic control. To address this bias, specific correction factors tailored to the disease have been recommended for diagnosis and treatment management (40). One well-accepted common mechanism leading to increased fracture risk in both T1DM and T2DM is reduced bone turnover, which reduces the number of Haversian remodeling units and increases tissue age with compromised tissue toughness. As the resolution limit of current *in-vivo* x-ray devices is well above the normal diameter of Haversian canals (20 – 50 µm) and no mechanical tissue properties are measured by means of x-ray absorption, these changes are not or only indirectly detectable with these technologies. Of note, the increased *aBMD* and *vBMD* levels measured by DXA and HR-pQCT (42), respectively, in DM patients can be partly explained by a reduced Haversian canal density in the cortical tissue compartments. The accumulation of very large intracortical pores (diameter > 100 µm), as observed in several HR-pQCT studies on patients with T2DM, indicates impaired tissue remodeling (43). HR-pQCT measurements, when combined with Finite-Element-Analyses (FEA), can estimate stiffness and failure loads; however, these models assume invariant tissue properties and thereby only reflect structural changes. For example, in a recent cross-sectional study, T2DM was associated with a higher vBMD and cortical thickness. In women, T2DM was additionally associated with a higher stiffness and failure load at the radius (42). In another study on obese men with T2DM, elevated stiffness levels combined with worsened trabecular microstructure have been observed at the tibia (44).

In this study, DXA data were consistent with previous findings, *i.e.*, patients with DM had normal or elevated *aBMD* levels and T-Scores, which were more pronounced in patients with T2DM. DXA values were much more affected by the prevalence of DM than by the prevalence of fractures. The fracture discrimination by DXA in the investigated cohort was moderate. In contrast to DXA measurements, the CortBS microstructure and viscoelastic parameters, except for mean backscatter and attenuation amplitudes, and the derived CortBS score were not affected by prevalence and type of diabetes but were significantly altered in patients with fractures. Even after adjusting for the diabetes impact on the DXA scores, the associations of prevalent fractures with DXA scores were lower than those with the CortBS scores. Consequently, the fracture discrimination performance of the CortBS score for all fracture types was better than that of any DXA score. This was also true for the CortBS score with anthropometric data compared to DXA with anthropometric data (p < 0.0001).

The reduced backscatter and attenuation amplitudes in DM patients are in line with the theory of reduced bone turnover that decreases the number of Haversian remodeling canals. During the normal bone remodeling process, osteoclasts create a canal with an average diameter of 200 µm through tissue resorption. The canal is then refilled by osteoblasts through tissue synthesis, leaving a vascularized Havers canal with a diameter of approximately 30 µm (19). The onset of T1DM typically develops during childhood or adolescence. This causes reduced bone turnover that results in delayed skeletal growth and a delayed accumulation of remodeling canals. Over time, the number of remodeling canals increases, eventually reaching a canal density equivalent to that in normal tissue. If tissue resorption and tissue formation rates are in balance, pore size morphology remains unchanged. These effects can be observed in the T1DM groups. T1DM patients without fractures were characterized by low pore density, which is indicated by low attenuation and backscatter amplitudes, and normal pore diameter distribution along with low porosity. T1DM patients with fractures showed pore density, porosity, and pore size distribution levels that were not different from controls without fractures. This suggests that the prevalence of fractures in T1DM patients is most likely associated with compromised tissue toughness, as suggested by Qian et al. (11). This hypothesis is further supported by a study of Novak et al. (45) that found that young women with T1DM had significantly decreased cortical bone strength strain index in the tibia measured by pQCT.

The onset of T2DM typically occurs in adulthood at advanced ages, a period during which normal bone remodeling would have likely resulted in a ballpark number of Haversian remodeling canals. Furthermore, age-related bone loss is associated with an adapted structural bone remodeling in long bones, i.e., bone is predominantly resorbed at the endosteal interface and deposited at the periosteal interface, leading to reduced cortical thickness and increased bone diameter. In healthy elderly individuals, this tissue adaptation is accompanied by increased bone turnover, characterized by normal pore density. However, in diabetes patients, including older adults, reduced bone turnover is expected to result in lower pore density. Our data suggests that the bone tissue structure in T2DM results from a reduction of pore density due to reduced turnover and an accumulation of larger pores due to impaired remodeling. Indeed, the largest change in porosity, from 6.5% in individuals without fractures to 12.1% in individuals with fractures, was observed among T2DM patients. These findings are corroborated by a recent study by Heilmeier et al. (16), who have monitored a cohort of postmenopausal women with T2DM over five years. Compared to patients without fractures and controls, patients with fractures show increased porosity, which increased at a rate similar to the controls over the five-year follow-up. In line with our findings, this suggests that there is a causal relationship between increased cortical porosity and fractures in patients with T2DM. Therefore, cortical porosity could be used as a biomarker for assessing fracture risk. Noteworthy, several HR-pQCT studies have not been able to confirm the increase in cortical porosity in T2DM, which undermines our hypothesis that elevated cortical porosity is a cause of increased fracture risk in T2DM. In a population-based study on elderly women with T2DM by Nilsson et al. (46), HR-pQCT measurements revealed lower bone material strength by micro indentation in combination with an overall better microstructure compared to controls. Nonetheless, since HR-pQCT is incapable of resolving small pores, the full picture of the bone microstructure in T2DM could not be previously determined.

### Quantitative bone ultrasound in individuals with diabetes

The application of QUS in DM patients, mostly T2DM patients, has yielded mixed results. To our knowledge, this is the first time a cortical pulse-echo device was used to study diabetic patients. Most devices have been applied in anatomic locations other than the tibia. Tao et al. (33) used an axial transmission device in postmenopausal women with T2DM at the tibia in addition to the phalanges and the radius. Lower *SOS* values and elevated *aBMD* values were found at all locations in the diabetic cohort. At the radius, they were also associated with disease duration. Lower QUS parameters have also been found by other QUS devices in diabetic patients. More recently, the application of REMS, which analyses the ultrasound backscatter of the trabecular tissue at the femoral neck and spine, revealed lower REMS-BMD and elevated DXA T-Scores in postmenopausal women with T2DM (37). Similar results were found in a similar cohort with a device that employs a cortical transverse transmission method at the phalanges. The longitudinal study found no difference between diabetic and control patients at baseline. Three years later, the Amplitude-dependent Speed Of Sound (Ad.SOS) and Bone Transmission Time (BTT) decreased. The authors attribute this finding to an increased cortical porosity, in line with our results, as well as to an increased trabecular density (47). The application of the same device in premenopausal women with T1DM yielded reduced QUS parameters (Ad.SOS, Ultrasound Bone Profile Index (USBPI), T-Score, Z-Score), which could be associated with poor glycemic control (48). Another longitudinal study has used this device on young women with T1DM. Both at baseline and 10 years later, the QUS parameters were lower compared to controls, but no significant decline was found when the baseline was compared to the 10-year follow-up (49). Another study in a cohort of T2DM and non-diabetic men (50) focused on investigating the impact of lifestyle factors on bone health. In both groups, a decrease in heel bone stiffness was observed with age, although the association with age was only significant in healthy controls. Apart from that, the only difference displayed by diabetic patients was a slightly lower stiffness index in the age group of 30-40.

Interestingly, some studies have shown unchanged or improved QUS parameters in diabetic patients. Sosa et al. (34) conducted a study with a transverse transmission QUS device at the calcaneus and DXA on postmenopausal, T2DM, and obese women and healthy controls. BMD values of the lumbar spine were elevated in diabetic patients, however, there were no differences in QUS parameters. Yamaguchi et al. (51) conducted a study to assess the risk of vertebral fractures in T2DM patients using a QUS device at the calcaneus (CM-200; Elk Corp., Osaka, Japan). Neither BMD values nor QUS parameters, such as calcaneal SOS, were associated with a prevalent vertebral fracture in T2DM. Dobnig et al. (36) applied calcaneus ultrasound as well as axial transmission at the phalanges and radius in nursing home female patients with T2DM. In diabetic patients, the QUS parameters (e.g., calcaneus stiffness, radial and phalangeal SOS) were elevated, together with lower osteocalcin and parathyroid hormone. In a more recent study by Lasschuit et al. (52), another heel QUS device (Cuba sonometer) was applied in men and women with T2DM. In the follow-up, both BMD and broadband ultrasound attenuation were increased in diabetic patients, and no increased risk of fracture could be observed. Discrimination performance by ROC for DXA and QUS parameters was similar across all groups. For example, BUA performed similarly to femoral neck BMD in predicting fractures (52). Almost all these studies have utilized devices at the calcaneus, which are known to perform similarly to DXA in fracture risk discrimination in non-diabetic cohorts (53). Conti et al. (54) assessed which factors correlated with calcaneus-QUS parameters (BUA, SI/QUI, estimated BMD), with special regard to diabetic complications, being the only study that applied a QUS device in a cohort of both T1DM and T2DM. The QUS parameters were similar in both DM types, although there was a slight reduction in the estimated BMD of T1DM patients. The main findings were an association between diabetic neuropathy and worse QUS parameters and increased QUS parameters with increasing BMI.

The calcaneus is mostly made up of trabecular bone, for which HR-pQCT studies have shown normal to improved microstructure in T2DM (28). The broadband ultrasound attenuation measured by QUS has been proposed to be more of a reflection of bone mass rather than microstructure (55), which potentially explains why the application of calcaneus devices in T2DM has shown improved or similar QUS parameters to DXA when compared to controls. Our CortBS results show moderate correlations to DXA parameters and improved fracture risk discrimination, which is inconsistent with the findings by Lasschuit et al. (52) and Yamaguchi et al. (51) using calcaneal QUS. Concerning the complexity of bone tissue microstructure alteration in patients with DM, it is reasonable to assume that not all QUS devices may be suited for improving fracture risk discrimination compared to DXA. While most bone QUS technologies measure acoustic properties, e.g. speed of sound and broadband ultrasound attenuation, which may be empirically associated with fragility fractures, CortBS measures for the first time intracortical microstructural and viscoelastic cortical tissue properties, which have previously been shown to provide superior fracture discrimination performance compared to DXA in postmenopausal women with low bone mineral density (39). This study confirmed that CortBS parameters are associated with prevalent fractures and revealed additional related to reduces bone turnover in both type 1 and type 2 diabetes.

Both type 1 and type 2 diabetes. The combination of multiple CortBS parameters by means of PLS discrimination models provided an improved fracture discrimination performance compared to DXA.

### Association of tibia properties with fractures at central skeletal sites

The relationship between tibial measurements and fracture risk at other sites may seem counterintuitive. However, pathological skeletal deterioration is systemic and is therefore reflected in different bones throughout the body, including bones less susceptible to osteoporotic fractures. Areal BMD measured by DXA at the femur and vertebra are well known to predict fractures beyond these specific anatomic sites. The FRAX tool, based on DXA, estimates the risk of major osteoporotic fractures, including those at the shoulder and wrist. Several studies using HR-pQCT report associations between cortical parameters of peripheral bones, including the tibia, with the prevalence of fractures or fracture risk at major sites (43, 56–66). Therefore, measuring cortical bone using ultrasound at the tibia may also reflect bone characteristics at less accessible sites like the femoral neck and vertebra. Indeed, Iori et al. have shown in ex-vivo studies that cortical thinning and the accumulation of large intracortical pores in the tibia midshaft is associated with structural deterioration of the femoral neck (67) and reduced femur strength (68). Moreover, in a previous pilot study CortBS parameters measured in postmenopausal women provided a superior discrimination performance for vertebral and non-vertebral fractures compared to DXA. Our findings are also in line with an ex-vivo study of Cirovic et al. (69), where biopsies from male donors with T2DM revealed higher cortical porosity (Ct.Po) and lower cortical thickness (Ct.Th). T2DM subjects also showed significant differences in the structure model index and lower cortical and trabecular bone microhardness compared to controls. These changes increase the risk of femoral neck fractures in T2DM. The deteriorated cortical microarchitecture and reduced bone microhardness suggest altered bone composition in the superolateral femoral neck, which is not detected by standard DXA measurements.

### Limitations

Several limitations should be noted. First, this study is cross-sectional, investigating prevalent, not incident fractures, regardless of fracture mechanisms (inadequate trauma *vs*. fragility fracture). This approach was necessary due to the overall low number of recorded vertebral fractures. Additionally, some asymptomatic vertebral fractures might have been missed, as we did not routinely perform a complete lateral vertebral assessment and morphometric analysis. Incomplete laboratory data prevented the analysis of bone turnover parameters in relation to cortical parameters by CortBS, limiting our ability to further support our hypothesis. Moreover, 93 screened diabetic patients were excluded due to a high BMI (> 35 kg/m²). Given the high prevalence of metabolic syndrome in this risk group, future studies are needed to investigate a population with unrestricted BMI for broader applicability. No real-time analysis of the CortBS measurement quality was possible in this study, which led to the exclusion of data from 5 subjects during the *post-hoc* data analysis. For clinical applications, the data quality assessment needs to be incorporated into the measurement, providing real-time feedback to the operator and the possibility to repeat the measurement, until an appropriate data quality is achieved. Finally, the study population was comparatively small for each diabetes type and heterogeneous regarding diabetes history and medication, introducing a risk of confounding, by e.g. underlying medication, bone turnover parameters, concomitant diseases and medication, and others. A potential measurement bias cannot be completely ruled out due to the lack of cross-calibration between the devices; however, this is likely minimal given the small number of patients with external scans. Future studies with larger sample size, more homogenous patient and control cohorts, a prospective study design, and an improved ultrasound data acquisition protocol, including an immediate data analysis after the measurement, should therefore be conducted to confirm our results.

## Conclusion

This study is the first to demonstrate that using quantitative ultrasound to measure tibial intracortical pore morphology and viscoelastic properties likely reflects a key underlying pathophysiology of diabetes-related bone disease. In particular, it is clinically meaningful for identifying diabetes patients at higher fracture risk, potentially overcoming the “diabetes fracture paradox”. Future longitudinal studies with larger diabetic populations will help further characterize its value in fracture prediction and clarify the (diabetes-specific) factors influencing cortical bone morphology over time. This approach holds significant potential for improving fracture prevention in diabetic patients using an easily accessible, radiation-free tool.

## Data Availability

The raw data supporting the conclusions of this article will be made available by the authors, without undue reservation.
