# Supplementary material for: Quantitative ultrasound imaging reveals distinct fracture-associated differences in tibial intracortical pore morphology and viscoelastic properties in aged individuals with and without diabetes mellitus – an exploratory study
Source: Front Endocrinol (Lausanne). 2024 Dec 16;15:1474546. doi: 10.3389/fendo.2024.1474546 (PMC11683365; doi:10.3389/fendo.2024.1474546)

**Supplementary Materials**

**TABLE A1** | Impacts of age, sex, anthropometric information, co-morbidities and medications on DXA and US parameters obtained by multivariate ANOVA or stepwise linear regression analyses. Significant associations are marked with an asterisk, for weight and height the directions of change are indicated by ‘+’ or ‘-’. Note that BMI was only selected by the stepwise regression, if weight and height were excluded. For all associations with categorial variables, the F-values are listed. Differences between controls, T1DM, and T2DM groups obtained from post-hoc multi-comparison tests are indicated by ‘<’.


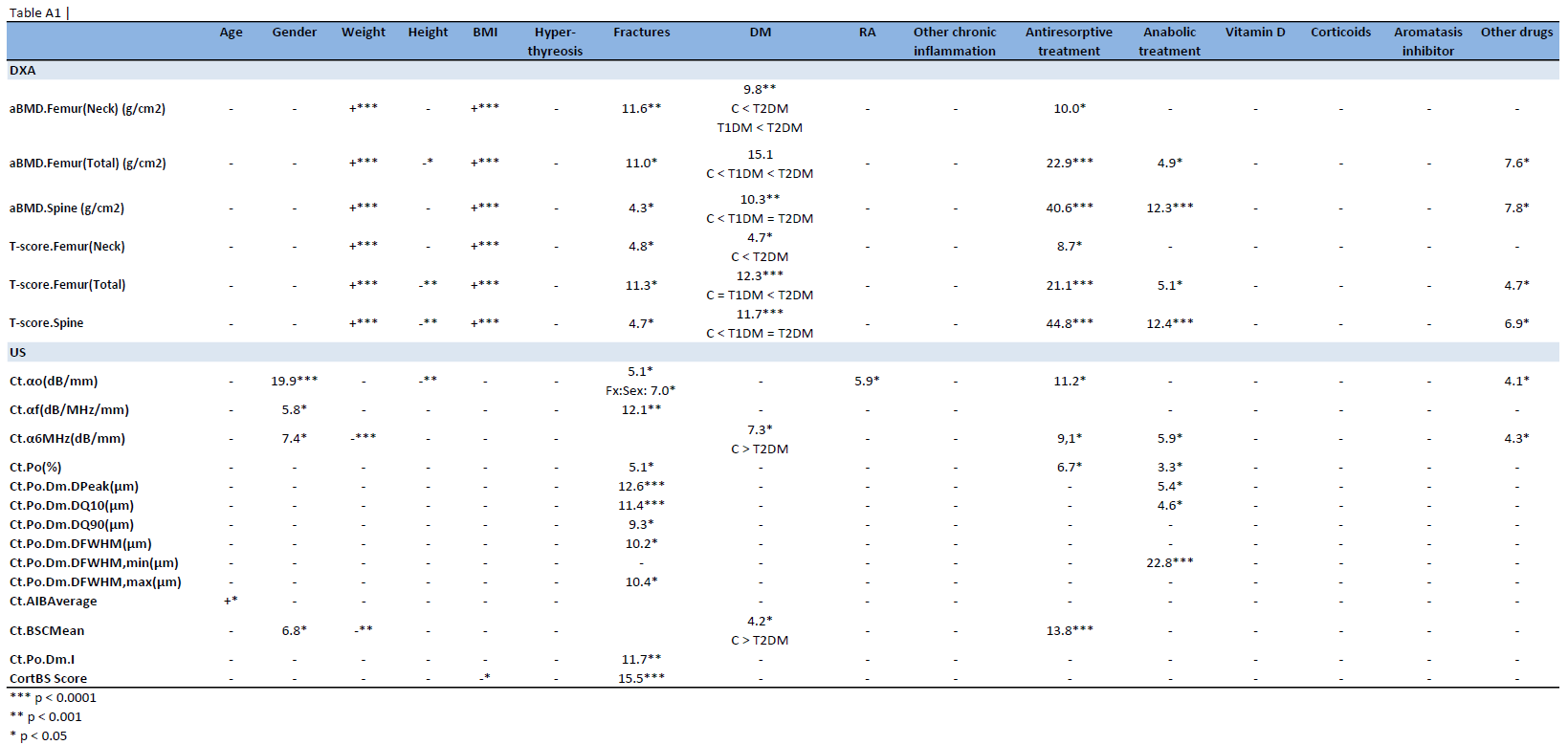

Supplement: Supplementary file 1 [file Table1.docx]
